# Supplementary material for: Abortion and contraception for incarcerated people: A scoping review
Source: PLoS One. 2023 Mar 30;18(3):e0281481. doi: 10.1371/journal.pone.0281481 (PMC10062621; doi:10.1371/journal.pone.0281481)
Supplement: S1 Appendix — (DOCX) [file pone.0281481.s001.docx]

### **Appendix 1: Search Strategy**

| **CINAHL** | | | Thursday, February 10, 2022 |  |  |
| --- | --- | --- | --- | --- | --- |
|  | **#** | **Query** | | | **Results** |
|  | S1 | MH "Prisoners") OR (MH "Correctional Facilities") OR (MH "Correctional Health Services") OR (MH "Correctional Health Nursing") | | | 15,159 |
|  | S2 | prison* OR incarcerat* OR correction* OR offender* OR penitentia* OR inmate* OR convict* OR jail* | | | 89,744 |
|  | S3 | S1 OR S2 | | | 89,744 |
|  | S4 | (MH "Abortion, Induced+") OR (MH "Abortifacient Agents") OR (MH "Carboprost") OR (MH "Mifepristone") OR (MH "Misoprostol") | | | 13,013 |
|  | S5 | TI ( abortifacient* OR abortion* OR (menstrua* W3 regulat*) OR pre‐abortion OR preabortion OR post‐abortion OR postabortion OR post‐abortum OR postabortum OR feticid* OR foeticid* OR ((medical* OR medication OR medicin* OR trimester* OR gestation* OR pregnan*) W5 (post‐terminat* OR postterminat* OR pre‐terminat* OR preterminat* OR terminat*)) ) OR AB ( abortifacient* OR abortion* OR (menstrua* W3 regulat*) OR pre‐abortion OR preabortion OR post‐abortion OR postabortion OR post‐abortum OR postabortum OR feticid* OR foeticid* OR ((medical* OR medication OR medicin* OR trimester* OR gestation* OR pregnan*) W5 (post‐terminat* OR postterminat* OR pre‐terminat* OR preterminat* OR terminat*)) ) | | | 13,977 |
|  | S6 | TI ( Mifepristone OR Misoprostol OR Abo‐pill OR Colestone OR Cytotec OR Elmif OR Epostane OR Fenprostalene OR GyMiso OR Korlym OR Medabon OR Mefeprin OR Mefipil OR Mifebort OR Mifegest OR Mifegyne OR Mifeprex OR Miferiv OR Mifty OR Mtpill OR Nalador OR RU‐38486 OR RU38486 OR RU‐486 OR RU486 OR T‐Pill OR Termipil ) OR AB ( Mifepristone OR Misoprostol OR Abo‐pill OR Colestone OR Cytotec OR Elmif OR Epostane OR Fenprostalene OR GyMiso OR Korlym OR Medabon OR Mefeprin OR Mefipil OR Mifebort OR Mifegest OR Mifegyne OR Mifeprex OR Miferiv OR Mifty OR Mtpill OR Nalador OR RU‐38486 OR RU38486 OR RU‐486 OR RU486 OR T‐Pill OR Termipil ) | | | 2,654 |
|  | S7 | (MH "Contraception+") OR (MH "Contraceptive Agents+") OR (MH "Contraceptive Devices+") | | | 41,971 |
|  | S8 | TI ( Contracept* OR (family N1 planning) OR (birth N1 control) OR condom OR (OC N1 pill) OR (depot medroxyprogest* or NET‐EN or NET EN or Mesigyna or Cyclofem) OR (intrauterine system or intra‐uterine system or IUS or intrauterine device or intra‐uterine device or IUD) OR OR (vasectomy or sterilisation or sterilization or (tubal N1 ligation)) OR OR ((vaginal N1 ring) or cycletel or cycle‐tel or abstain or abstinen* or lactational amenorr*) ) OR AB ( Contracept* OR (family N1 planning) OR (birth N1 control) OR condom OR (OC N1 pill) OR (depot medroxyprogest* or NET‐EN or NET EN or Mesigyna or Cyclofem) OR (intrauterine system or intra‐uterine system or IUS or intrauterine device or intra‐uterine device or IUD) OR OR (vasectomy or sterilisation or sterilization or (tubal N1 ligation)) OR OR ((vaginal N1 ring) or cycletel or cycle‐tel or abstain or abstinen* or lactational amenorr*) ) | | | 35,606 |
|  | S9 | S4 OR S5 OR S6 OR S7 OR S8 | | | 72,106 |
|  | S10 | S3 AND S9 | | | 770 |
