# Supplementary material for: Abortion and contraception for incarcerated people: A scoping review
Source: PLoS One. 2023 Mar 30;18(3):e0281481. doi: 10.1371/journal.pone.0281481 (PMC10062621; doi:10.1371/journal.pone.0281481)
Supplement: Attachment — Submitted filename: PLOS One 11_13.docx [file pone.0281481.s003.docx]

Abortion and Contraception for Incarcerated People: A Scoping Review

Manuscript Number: PONE-D-22-27437

The authors present a study looking at the landscape of contraception and abortion access for individuals who have been incarcerated. Notably, the authors performed a global review of the literature (geographically expansive), and included a reproductively diverse study population (all people who have uteruses).

Introduction

- The authors concisely highlight the need for more information regarding contraception and abortion for incarcerated people
- The introduction references research previously done in the US and changes to US policies regarding abortion access (ie Dobbs), but asserts the importance of this study is the international lens. It may strengthen the introduction to add some information regarding the impact of incarceration on sexual and reproductive health care for international poplations as well
  - There is some explanation on why the US predominates the literature in the discussion section, but may be beneficial to address the international need earlier

Methods

- No issues

Results

- No issues

Discussion

- The authors do an excellent job of discussing the results within the Levesque definition of health service access and with the lens of abolition feminism. While reading, there were a few questions that came to mind that may be of interest for the authors to discuss further?
  - Approachability speaks to the relationship between patients/providers. Although only one study (Sufrin, et al) addresses the experience of the providers, it may be interesting to juxatpose the provider barriers with the patient reported barriers to further explore some of the issues further. What makes it hard for HCPs to connect with patients in the carceral setting vs community setting? Did any of the studies explore this?
    - Highlighting the significance of COs in this dynamic was important. Well done.
  - Affordability: This topic can be addressed on an individual and systems level.
    - Did you find in your review that many institutions were charging patients for contracpetive methods while incarcerated? I didn’t see that in the results. This paragraph seemed to suggest the cost of the method to patients was major influence on contraceptive choice and access while incarcerated. I understand the point being made, but fear it may be misrepresented.
    - It may be interesting to discuss how the cost of contraceptive methods affect which medications are kept on formulary and/or how this may be an area of further exploration.
    - This is also a good place to discuss the impact insurance status on access in the community setting to contraception and abortion. Discuss geographical variances.
  - The authors appropriately discuss acceptability as an issue among this population. I encourage the authors to explore some of the available literature regarding social determinants of health for incarcerated individuals and consider the interplay on sexual and reproductive health.
- I would add to the discussion the dearth of knowledge around contraception for individuals in the LGBTQ+ community and how the findings in this study can deeply impact their experience both while incarcerated and in the community (especially as they were added to the study population intentionally)
  - It is discussed in the limitations, but I would consider this a finding and highlight the importance of research geared towards this community

Overall, I thought the authors explored this topic well and used appropriate frameworks to contexutalized the information.
