# Supplementary material for: Abortion and contraception for incarcerated people: A scoping review
Source: PLoS One. 2023 Mar 30;18(3):e0281481. doi: 10.1371/journal.pone.0281481 (PMC10062621; doi:10.1371/journal.pone.0281481)
Supplement: Attachment — Submitted filename: PlosOne responses to reviewers 20230123.docx [file pone.0281481.s005.docx]

Responses to Reviewers

Abortion and Contraception for Incarcerated People: A Scoping Review

Manuscript Number: PONE-D-22-27437

| Comment | Response |  |
| --- | --- | --- |
|  |  |  |
| 1. Many authors now use "criminal legal system" instead of "justice system" to reflect how little justice there is in the system. Please consider making this change. | We have made this change throughout the manuscript. |  |
| 2. Similarly, "carceral facility" or a more specific term such as "detention center" or "jail" is preferred over "correctional facility" due to the paucity of corrections that occur in these facilities. | We have made this change throughout except when referencing “correctional officers/staff” as this is their job title. |  |
| 3. "Unclothed body searches" is preferred to "strip searches." | Changed, thank you. |  |
